# Changes of retinal flow volume after intravitreal injection of bevacizumab in branch retinal vein occlusion with macular edema: a case series

**DOI:** 10.1186/s12886-016-0239-8

**Published:** 2016-05-25

**Authors:** Hidetaka Noma, Kanako Yasuda, Terumi Minezaki, Sho Watarai, Masahiko Shimura

**Affiliations:** Department of Ophthalmology, Hachioji Medical Center, Tokyo Medical University, 1163, Tatemachi, Hachioji, Tokyo 193-0998 Japan

## Abstract

**Background:**

Although intravitreal injection of bevacizumab (IVB) is effective for macular edema in patients with branch retinal vein occlusion (BRVO), the changes of retinal hemodynamics remain unclear. We investigated retinal hemodynamic changes in BRVO patients after IVB by performing laser speckle flowgraphy (LSFG).

**Methods:**

In 35 BRVO patients with macular edema, the relative flow volume (RFV) of the retinal artery and vein passing through the optic disc was measured in both the occluded and non-occluded regions of the retina before IVB and 1 month after IVB by LSFG. The ischemic region of retina was measured with the Scion Image program and the severity of retinal ischemia was assessed by dividing the non-perfused area by the disc area.

**Results:**

Macular edema improved significantly by 1 month after IVB. The venous RFV ratio showed a significant increase in the non-occluded region at 1 month after IVB. There was a significant negative correlation between the venous RFV ratio and the severity of retinal ischemia in the occluded region. On the other hand, arterial RFV ratio showed no significant change after IVB in either the occluded or non-occluded region. In addition, there was no significant correlation between the arterial RFV ratio and the severity of retinal ischemia in either the occluded or non-occluded region.

**Conclusions:**

These results suggest that an increase of retinal venous outflow after IVB may possibly influence the resolution of macular edema and that the response of venous outflow after IVB depends on the severity of retinal ischemia in the occluded region.

## Background

Branch retinal vein occlusion (BRVO) is a very common retinal vascular condition in patients with lifestyle-related diseases such as hypertension and arteriosclerosis. Because retinal arterioles and venules have a common adventitia at arteriovenous crossings, the vein walls can be compressed by arteriosclerotic changes. Luminal narrowing leads to disturbance of laminar blood flow and endothelial damage is caused by shear stress, after which thrombus formation leads to BRVO [1]. In the acute phase of BRVO, pressure increases in the capillaries and venules affected by obstruction, leading to breakdown of the blood-retinal barrier and leakage of blood components. The main cause of visual impairment in BRVO patients is macular edema. Thus, understanding the retinal hemodynamic abnormalities that underlie the pathogenesis of BRVO is of critical importance.

A multicenter study [2] has shown that intravitreal injection of bevacizumab (IVB) is effective for macular edema in BRVO patients, but the retinal hemodynamic changes remain unclear. Laser speckle flowgraphy (LSFG) is a noninvasive technique based on the laser speckle phenomenon that allows simultaneous assessment of blood flow in the vessels of the optic nerve head, choroid, and retina [3]. The relative flow volume (RFV) obtained with LSFG was recently reported to be an accurate and reliable index of blood flow volume in the retinal vessels [4]. This index could potentially provide an accurate assessment of retinal blood flow in the superficial layer of the retina [4].

In the present study, changes of retinal hemodynamics after IVB were investigated in BRVO patients with macular edema by LSFG. RFV was measured separately for retinal arteries and veins in the occluded and non-occluded regions of the retina.

## Methods

### Subjects

This study was conducted at the Department of Ophthalmology of Tokyo Medical University and approval was obtained from the University Ethics Committee. The procedures of the study conformed to the tenets of the Declaration of Helsinki and all patients gave written informed consent before enrollment. Thirty-five patients with BRVO (35 eyes) who were scheduled to undergo intravitreal injection of bevacizumab (1.25 mg in 0.05 ml, Avascin; Genentech and Hoffmann La Roche, Basel, Switzerland) were studied between January and August 2013. Criteria for receiving IVB were macular edema involving the fovea (central macular thickness (CMT) > 300 μm), and best-corrected visual acuity (BCVA) < 20/30. There were 18 men and 17 women aged 67.6 ± 11.0 years (mean ± SD). The mean duration of macular edema was 53.3 ± 40.4 days (range: 15–180 days). Twenty-three of the 35 patients (66 %) had hypertension and 15 of the 35 patients (43 %) had hyperlipidemia.

Exclusion criteria were glaucoma, aphakia, rubeosis iridis, clinically significant cataract, ocular infection, a history of retinal diseases other than BRVO, laser coagulation, refractive error of less than - 6.0 diopters, and intraocular surgery (including cataract surgery) on the index eye within 6 months of the scheduled time for injection of bevacizumab.

### Routine examinations

At the initial visit, all patients underwent a complete ophthalmic examination including decimal BCVA, fluorescein angiography (FA) (Digital Retinal Camera CF-1; Canon, NY, USA) and spectral-domain OCT (Spectralis, Heidelberg Engineering, Heidelberg, Germany). A masked grader independently assessed retinal ischemia by examining the angiograms and measuring the ischemic region of the retina with the public domain Scion Image program [5]. On digital fluorescein photographs, the disc area was outlined with a cursor and then measured, after which the same was done for the non-perfused area. The severity of retinal ischemia was assessed as the non-perfused area divided by the disc area. For follow up, BCVA was determined and OCT was performed at 1 month after IVB. The CMT was defined as the distance between the inner limiting membrane and the retinal pigment epithelium (including any serous retinal detachment), and was automatically measured by computer software. BCVA was converted to the logarithm of minimal angle of resolution (logMAR) scale for statistical analysis.

### Laser speckle flowgraphy (LSFG)

LSFG (Softcare, Fukutsu, Japan) has been described in detail previously [3]. In brief, a fundus camera equipped with a diode laser (wavelength, 830 nm) and a charge-coupled device sensor (750 × 360 pixels) are used to obtain images of the pattern of speckle contrast produced by interference as laser light is scattered by red blood cells moving through vessels in the ocular fundus. Light reflected from the tissue produces a speckled pattern on the plane where the area sensor is focused and reflected light from moving erythrocytes causes blurring of the speckle pattern [6].

The mean blur rate (MBR) was calculated from variation of the blurring as a quantitative index of relative blood flow velocity expressed in arbitrary units (AU) [4, 7]. Images were acquired continuously at the rate of 30 frames/sec over a 4-sec period and then averaged to produce a composite map of ocular blood flow.

**Fig. 1:**
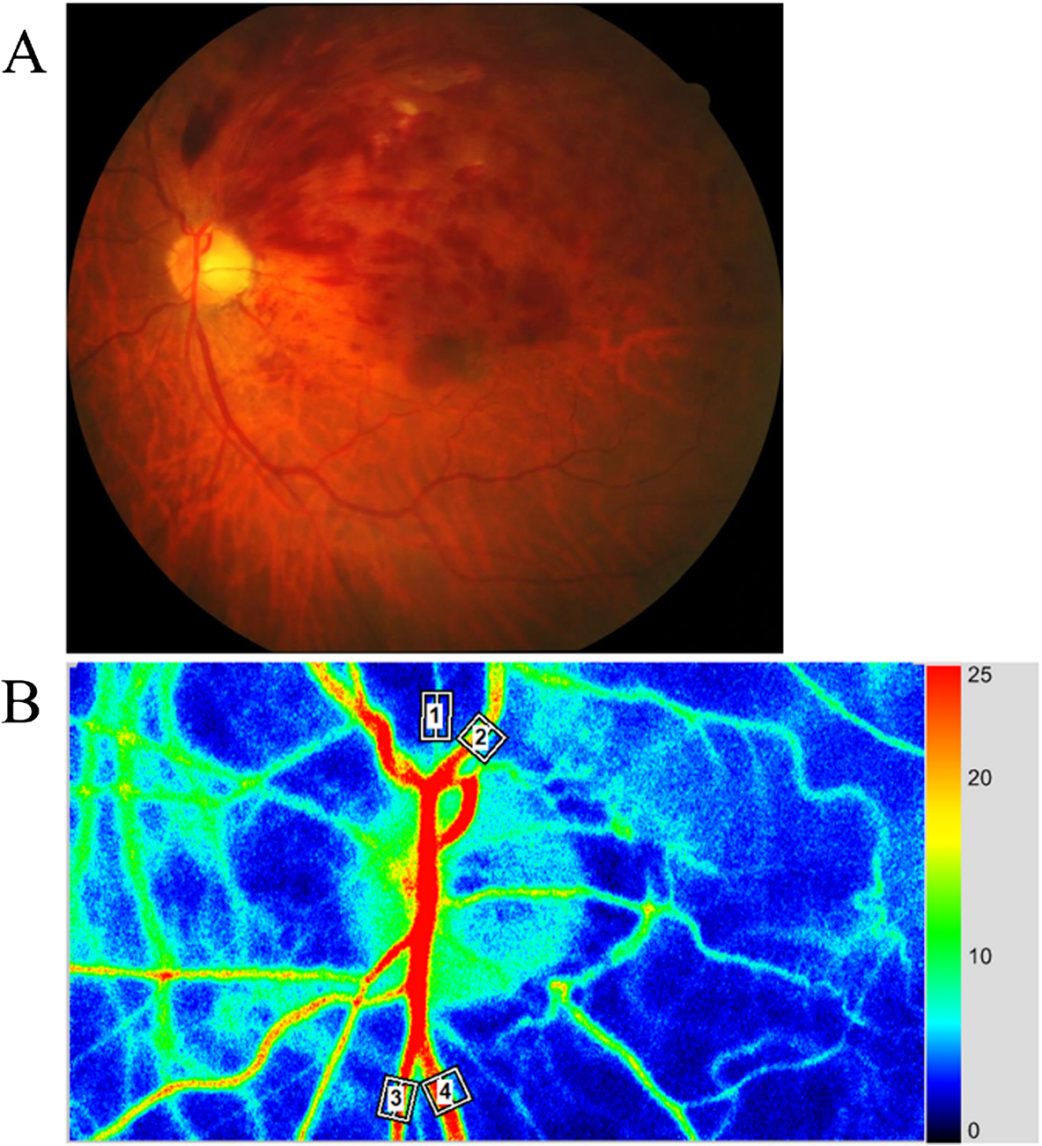
Representative fundus color photograph and representative RFV data obtained with LSFG. **a** Fundus color photograph shows branch retinal vein occlusion (BRVO). The upper part is the occluded region and the lower part is the non-occluded region. **b** Blood flow in an artery (white square #1) and a vein (white square #2) from the occluded region was automatically tracked in the images. Blood flow in an artery (white square #3) and a vein (white square #4) from the non-occluded region was also automatically tracked

RFV was obtained by manually selecting a region of interest centered on a retinal vessel. A separate MBR value was then automatically calculated for the vessel by subtracting the background choroidal blood flow from the overall MBR value. Then RFV was determined by integrating the MBR values in a direction transverse to the retinal vessel. RFV reflects blood flow volume rather than flow velocity based on the relation between a trunk vessel and its branches [4], and it was calculated by using Cross-Section Ex in the LSFG analyzer software (version 3.1.6). The RFV of the artery and vein passing through the optic disc was measured in both the occluded and non-occluded regions before IVB and 1 month after IVB (Fig. 1a and b).

The pupil was dilated with 0.5 % tropicamide and 0.5 % phenylephrine hydrochloride 20 min before LSFG. To evaluate changes of RFV, the RFV ratio was calculated as a percentage of the respective baseline value (100 %), since these are qualitative indices of ‘relative’ blood flow.

### Hemodynamics

Within a certain range, the relationship between RFV and ocular perfusion pressure (OPP) is bilinear in healthy subjects with normal eyes [8]. To exclude physiological responses from the present results, the blood pressure and intraocular pressure (IOP) were measured to calculate the OPP. Mean blood pressure (MBP) was calculated from the systolic blood pressure (SBP) and diastolic blood pressure (DBP) as MBP = DBP + 1/3(SBP − DBP). Then OPP was calculated by using the following equation: OPP = 2/3MBP − IOP [9].

### Statistical analysis

All analyses were performed with SAS System 9.3 software (SAS Institute Inc., Cary, North Carolina, USA). Results are presented as the mean ± SD. Student’s *t*-test was employed to compare unpaired continuous variables with a normal distribution. The one sample *t*-test was used to assess the hypothesis that a mean value is equal to 100. Two-tailed p values of less than 0.05 were considered to indicate a significant difference.

## Results

The clinical characteristics of the BRVO patients are summarized in Table 1. There were 18 men and 17 women aged 67.6 ± 11.0 years (mean ± SD). The mean duration of macular edema was 53.3 ± 40.4 days (range: 15–180 days). Twenty-three of the 35 patients (66 %) had hypertension and 15 of the 35 patients (43 %) had hyperlipidemia.

**Table 1:** Clinical and demographic data of the BRVO patients

No. (Male/Female)	35 (18/17)
Age (yrs)	67.6 ± 11.0
Hypertension	23 (66 %)
Systolic Blood pressure (mmHg)	131 ± 17
Diastolic Blood pressure (mmHg)	78 ± 16
OPP (mmHg)	
Baseline	51.1 ± 8.5
1 month after IVB	51.2 ± 7.6
Hyperlipidemia	15 (43 %)
Duration of BRVO (days)	53.3 ± 40.4
Severity of retinal ischemia (disc area)	26.1 ± 30.1
BCVA (log MAR)	
Baseline	0.62 ± 0.34
1 month after IVB	0.29 ± 0.27
CMT (μm)	
Baseline	634 ± 235
1 month after IVB	286 ± 117

*BRVO* branch retinal vein occlusion, *No*. number of eyes, *OPP* ocular perfusion pressure, *BCVA* best-corrected visual acuity, *log MAR* logarithm of minimal angle of resolution, *IVB* intravitreal injection of bevacizumab, *CMT* central macular thickness

At the initial examination, the mean BCVA was log MAR 0.62 ± 0.34, and it improved significantly to log MAR 0.29 ± 0.27 by 1 month after IVB (p < 0.001) (Table 1). The initial mean CMT was 634 ± 235 μm and it decreased significantly to 286 ± 117 μm by 1 month after IVB (*p* < 0.001) (Table 1).

**Fig. 2:**
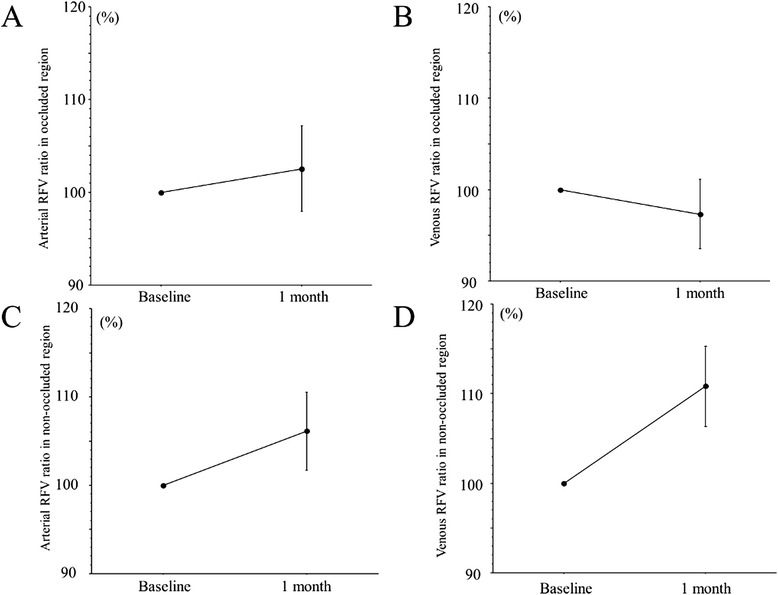
RFV ratios (arterial (**a**) and venous (**b**) in the occluded region or arterial (**c**) and venous (**d**) in the non-occluded region). **a** There was no significant change of the arterial RFV ratio (102.5 %) in the occluded region at 1 month after IVB (*p* = 0.586). **b** There was no significant change of the venous RFV ratio (97.3 %) in the occluded region at 1 month after IVB (*p* = 0.483). **c** There was no significant change of the arterial RFV ratio (106.1 %) in the non-occluded region at 1 month after IVB (*p* = 0.175). **d** The venous RFV ratio (110.8 %) in the non-occluded region showed a significant increase at 1 month after IVB (*p* = 0.021)

The RFV measured with LSFG are summarized in Table 2. Figure 2 (a-d) shows the RFV ratios in arterial and venous for the occluded and non-occluded regions.

**Table 2:** RFV (arterial and venous) in the occluded and non-occluded regions

	Occluded Region		Non-occluded Region	
Variable	Artery (A.U.)	Vein (A.U.)	Artery (A.U.)	Vein (A.U.)
Baseline	192 ± 113	287 ± 144	208 ± 93	355 ± 149
1 month after IVB	194 ± 112	280 ± 149	228 ± 107	393 ± 187

*RFV* relative flow volume, *IVB* intravitreal injection of bevacizumab

The RFV measured with LSFG changed from 192 ± 113 AU to 194 ± 112 AU, from 287 ± 144 AU to 280 ± 149 AU, from 208 ± 93 AU to 228 ± 107 AU, and from 355 ± 149 AU to 393 ± 187 AU, respectively (Table 2). The four RFV ratios were 102.5 %, 97.3 %, 106.1 %, and 110.8 % at 1 month after IVB, respectively (Fig. 2a-d). There was no significant difference of three ratios (arterial or venous of the occluded region and arterial of the non-occluded region) at 1 month after IVB (*p* = 0.586, *p* = 0.483, and *p* = 0.175, respectively) (Fig. 2a-c). However, the venous RFV ratio of the non-occluded region was significantly increased at 1 month after IVB (*p* = 0.021) (Fig. 2d).

**Fig. 3:**
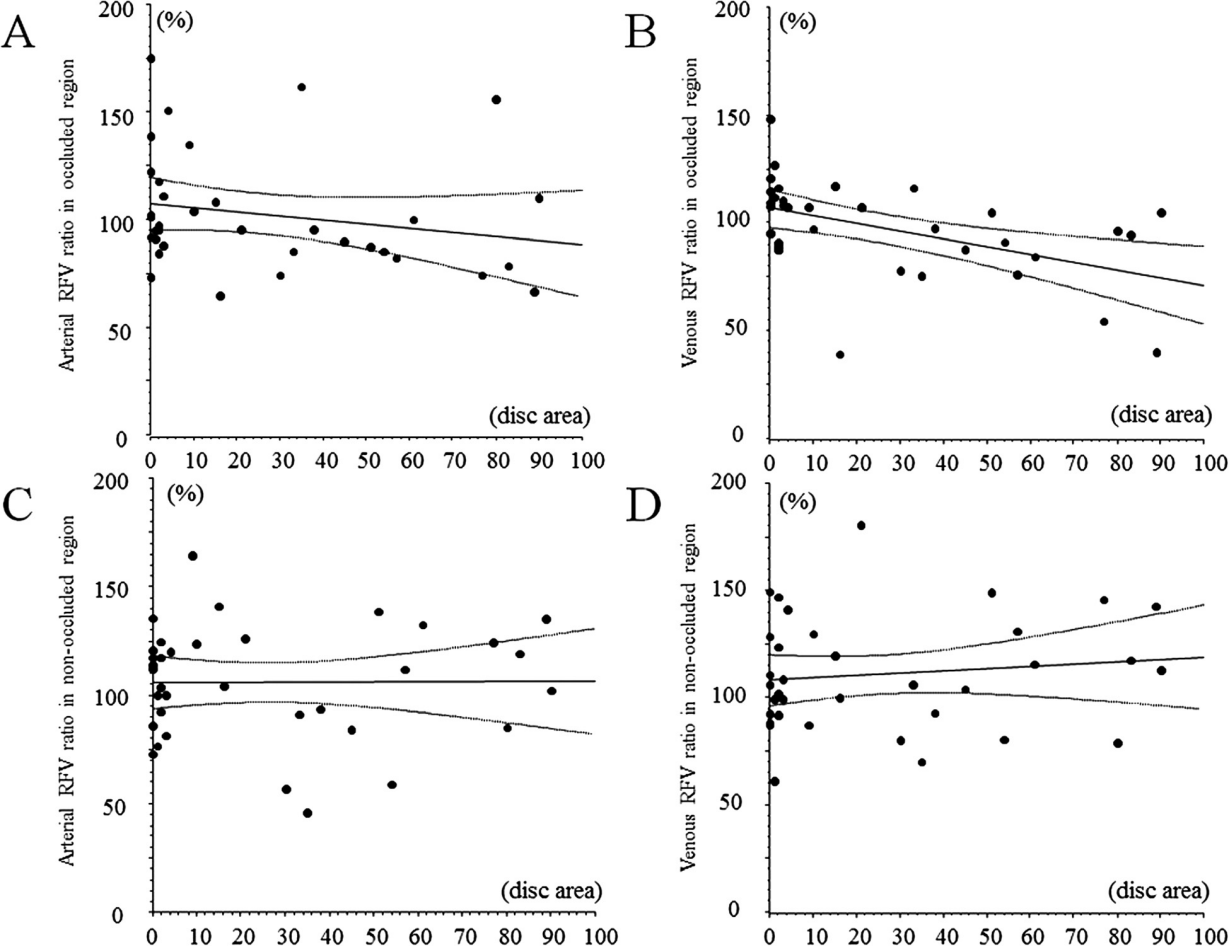
Correlation between the RFV ratio and the severity of retinal ischemia. **a** No significant correlation between the arterial RFV ratio and the severity of retinal ischemia in the occluded region (*r* = -0.21, *p* = 0.228). **b** Significant negative correlation between the venous RFV ratio and the severity of retinal ischemia in the occluded region (*r* = -0.49, *p* = 0.003). **c** No significant correlation between the arterial RFV ratio and the severity of retinal ischemia in the non-occluded region (*r* = 0.01, *p* = 0.988). **d** No significant correlation between the venous RFV ratio and the severity of retinal ischemia in the non-occluded region (*r* = 0.12, *p* = 0.481)

When the relationship between the RFV ratio and the severity of retinal ischemia was investigated, 2 subjects were excluded because assessing the non-perfused area was difficult due to severe retinal hemorrhage. A significant negative correlation was found between the venous RFV ratio and the severity of retinal ischemia in the occluded region (*r* = -0.49, *p* = 0.003) (Fig. 3b). On the other hand, there was no significant correlation between the other RFV ratios and the severity of retinal ischemia (arterial RFV ratio in the occluded region, arterial RFV ratio in the non-occluded region and venous RFV ratio in the non-occluded region: *r* = -0.21, *p* = 0.228, *r* = 0.01, *p* = 0.988, and *r* = 0.12, *p* = 0.481, respectively) (Fig. 3a, c, and d).

When BRVO eyes were divided into ischemic and non-ischemic group, this study included 14 ischemic eyes. In the ischemic group, there was a significant decrease in the venous RFV ratio of the occluded region after IVB (83.0 %, *p* = 0.006). In contrast, the other three RFV ratios (arterial ratio of the occluded region and both ratios of the non-occluded region) did not change significantly after IVB (95.0 %, *p* = 0.155, 102.6 %, *p* = 0.478, and 114.3 %, *p* = 0.474, respectively).

The OPP and ocular hemodynamics were measured in all eyes before and after IVB. There was no significant difference of OPP between before IVB (51.1 ± 8.5) and after IVB (51.2 ± 7.6) (*p* = 0.930) (Table 1). There was also no significant correlation between RFV and OPP, indicating that the observed changes of RFV were not influenced by the systemic circulation (data not shown). Furthermore, there were no significant correlations between the duration of macular edema and the four baseline RFV values (arterial or venous RFV of the occluded and non-occluded regions) (*r* = -0.18, *p* = 0.298, *r* = 0.01, *p* = 0.984, *r* = -0.10, *p* = 0.557, and *r* = -0.16, *p* = 0.354, respectively).

## Discussion

The present study revealed changes of the RFV in either the occluded or non-occluded region passing through the optic disc after IVB in BRVO patients with macular edema. First, we found that the venous RFV ratio only increased significantly in the non-occluded region at 1 month after IVB (Fig. 2d). This suggests that an increase of venous outflow in the non-occluded region removes fluid from the macular region and may influence the resolution of macular edema after IVB. Alternatively, resolution of macular edema may be primarily due to reduced vascular leakage because of the effects of anti-VEGF therapy rather than due to an increase of venous outflow in the non-occluded region.

Interestingly, we only found a significant negative correlation between the venous RFV ratio and the severity of retinal ischemia in the occluded region (Fig. 3b), suggesting that the response of venous outflow to IVB may depend on the severity of retinal ischemia in the occluded region alone. Generally, retinal ischemia is associated with capillary loss. Capillaries contribute to the regulation of blood flow in the retina through contraction or relaxation of pericytes, which are cells surrounding the capillaries that are similar to vascular smooth muscle cells [10]. Accordingly, capillary loss may have led to the significant decrease of venous RFV in the occluded region after IVB. In addition, the level of vascular endothelial growth factor (VEGF) in the vitreous fluid is correlated with the nonperfused retinal area in BRVO patients [11]. VEGF has been shown to stimulate the production of nitric oxide (NO), an endothelium-derived relaxing factor [12]. The endothelium of blood vessels releases NO to promote the relaxation of vascular smooth muscle, resulting in vasodilation and increased blood flow [13]. Therefore, a decrease of VEGF after IVB may lead to vasoconstriction secondary to reduced production of NO, and this may also have influenced the significant decrease of the venous RFV ratio in the occluded region after IVB. Recently, Shimura et al. reported that macular ischemia occurred after IVB in patients with central retinal vein occlusion [14]. Based on our finding that there was a significant negative correlation between the venous RFV ratio and the severity of retinal ischemia in only the occluded region, it is possible that IVB promoted ischemia by reducing RFV in the BRVO patients. Therefore, use of IVB in ischemic BRVO may need careful attention, and measuring RFV by LSFG-NAVI before treatment may be useful in BRVO patients. However, further investigations will be needed to clarify the RFV by using LSFG-NAVI in BRVO patients receiving treatment for macular edema.

The present study also demonstrated that there was no significant change of the arterial RFV ratio in either the occluded or non-occluded region at 1 month after IVB (Fig. 2a and c), and that there was no significant correlation between the arterial RFV ratio and the severity of retinal ischemia in either region (Fig. 3a and c). These results suggest that IVB does not influence the arterial blood supply of the retina and probably does not promote retinal ischemia. Autoregulation of retinal blood flow involves interaction of myogenic and metabolic mechanisms through the release of vasoactive substances by the vascular endothelium and retinal tissues surrounding the arterioles [15]. Autoregulation adapts the tone of the resistance vessels (arterioles and capillaries) to changes of the OPP or tissue metabolic requirements. This adaptation occurs through the interaction of multiple mechanisms affecting the arteriolar smooth muscle cells and capillary pericytes. Mechanical stretch and an increase of arteriolar transmural pressure induce the release of contractile factors affecting the tone of arteriolar smooth muscle cells and pericytes [15]. Nagaoka et al.[16] reported that the retinal arterioles play an important role in autoregulation of retinal blood flow via a myogenic mechanism. Thus, autoregulation of the retinal arterioles may ensure that there is no change of the arterial RFV ratio after IVB. In addition, development of macular edema in BRVO patients has been hypothesized to be caused by fluid flux from the vascular compartment to the tissues (Starling’s Law) [17, 18] after breakdown of the blood-retinal barrier. Taken together with the present findings, such reports suggest that an increase of retinal venous outflow rather than increased arterial blood supply may be important for improvement of macular edema by IVB.

The present study had several limitations. One major drawback was the lack of a control group. Second, this study demonstrated a significant increase of the venous RFV ratio in the non-occluded region after IVB, but this may have been a compensatory response like development of collateral vessels in the course of BRVO. Therefore, it is difficult to conclude that the increase of RFV in non-occluded veins was due to IVB. Accordingly, future studies comparing retinal venous flow in different eyes with BRVO immediately prior to treatment might provide useful data about the relative influence of bevacizumab therapy versus the natural course of the disease.

## Conclusions

In BRVO patients, macular edema demonstrated significant improvement by 1 month after IVB. The venous RFV ratio showed a significant increase in the non-occluded region at 1 month after IVB and there was a significant negative correlation between this ratio and the severity of retinal ischemia in the occluded region. On the other hand, the arterial RFV ratio did not change significantly in the occluded or non-occluded regions and there was no significant correlation between the arterial RFV ratio and the severity of retinal ischemia in either region. These results suggest the possibility that an increase of retinal venous outflow after IVB influences the resolution of macular edema and that the response of venous outflow to IVB depends on the severity of retinal ischemia in the occluded region.

## Abbreviations

AU, arbitrary units; BCVA, best-corrected visual acuity; BRVO, branch retinal vein occlusion; CMT, central macular thickness; DBP, diastolic blood pressure; FA, fluorescein angiography; IOP, intraocular pressure; IVB, intravitreal injection of bevacizumab; logMAR, logarithm of minimal angle of resolution; LSFG, laser speckle flowgraphy; MBP, mean blood pressure; MBR, mean blur rate; NO, nitric oxide; OCT, optical coherence tomography; OPP, ocular perfusion pressure; RFV, relative flow volume; SBP, systolic blood pressure; VEGF, vascular endothelial growth factor.
